# Effects of inactivated COVID-19 vaccination on HIV viremia and reservoir size: a longitudinal cohort study

**DOI:** 10.1186/s12879-025-12184-8

**Published:** 2025-12-13

**Authors:** Jie Li, Caiping Guo, Ruolei Xin, Yuchuan Deng, Can Pang, Jingrong Ye, Jia Li, Hongyan Lu, Xiaoxin He, Quanyi Wang

**Affiliations:** 1 Department of Epidemiology and Biostatistics, School of Public Health, Capital Medical University, Beijing, China; 2 Institute for HIV/AIDS and STD Prevention and Control, Beijing Center for Disease Prevention and Control, No.16, Hepingli Middle Road, Dongcheng District, Beijing, 100013 People’s Republic of China; 3 Department of Infectious Diseases and Medical Immunology, Beijing Youan Hospital, Capital Medical University, Beijing, China

**Keywords:** HIV, COVID-19, Inactivated vaccine, Plasma viral load, HIV-1 DNA

## Abstract

**Background:**

Vaccination is regarded as the most effective and cost-efficient mean of managing COVID-19. Whether receiving inactivated vaccine leads to plasma viral load (pVL) rebound and affects HIV reservoirs size has been a major concern for people living with HIV (PLWH). In this study we performed a longitudinal observational study to explore the dynamic changes of pVL and HIV-1 total DNA with PLWH after vaccination with inactivated COVID-19 vaccine.

**Methods:**

Information and venous blood samples from PLWH were collected prevaccination (BC1), three weeks after the first vaccination (BC2), four weeks after the second dose (BC3), six months after the second dose (BC4), and two weeks after the third dose (BC5) to test RBD-specific IgG antibody, plasma viral load (pVL), HIV-1 total DNA and CD4+ T cell count.

**Results:**

A total of 25 PLWH participated in this study, with a median age of 34 (IQR 28.5 − 40.0) years. No significant difference in proportion of undetectable pVL group, pVL ≥ 20 cp/ml group and pVL < 20 cp/ml group was observed among five time points (*p* = 0.506). Significant difference was observed in total HIV-1 DNA copies among different time points in both group of CD4+ T cells ≤ 300 and > 300. In the group of nadir CD4+ T cells > 300, pairwise comparison of five sets of data showed that total HIV-1 DNA copies at BC5 was significantly lower than BC1 (*P* = 0.043) and BC3 (*P* = 0.008). And duration of HIV infection was positively correlated with HIV-1 DNA copies at BC4 (*R* = 0.729, *p* = 0.007) and BC5 (*R* = 0.690, *p* = 0.013), S/CO value of RBD-specific-IgG at BC3 were negatively correlated with HIV-1 total DNA copies at time points of BC2 (*R*=-0.713, *p* = 0.009) and BC3 (*R*=-0.587, *p* = 0.045).

**Conclusions:**

Receiving inactivated COVID-19 vaccine didn’t significantly affect pVL. HIV-1 total DNA copies had a downward trend after vaccination. Duration of infection and IgG titer might be correlated with HIV-1 total DNA copies after vaccination.

**Supplementary Information:**

The online version contains supplementary material available at 10.1186/s12879-025-12184-8.

## Background

COVID-19 has become a very common disease affecting all ages of people since 2020. Vaccination is regarded as the most effective and cost-efficient mean for preventing severe outcomes of COVID-19. Administration of inactivated vaccines has been shown to generate a robust immune response [1].

HIV infection may increase the risk of be susceptible to various infections and elevate the risk of getting severe coronavirus disease 2019 (COVID-19) or even death. As a result, it was particularly important for PLWH to be vaccinated. Lots of evidence has showed that people living with HIV (PLWH) generally could produce protective immune responses to COVID-19 vaccination just as HIV-uninfected people did [1–3]. However, whether receiving inactivated vaccine leads to plasma viral load (pVL) rebound and affects HIV reservoirs size has been a major concern for PLWH.

Latent viral reservoir was recognized as a main obstacle to eliminate HIV-1 infection. Simonetti et al. [4] found antigen-driven proliferation could increase HIV-infected CD4+ T cells number in vivo, and clonal expansion of CD4+ T cells carrying intact proviruses contributed to the HIV-1 transcription, leading to transient increases in plasma HIV RNA levels [4, 5]. Therefore, understanding how COVID-19 vaccines lead to immune activation and affect HIV reservoirs is urgent and important. Inactivated COVID-19 vaccine could induce neutralizing antibody responses, elicit immune responses mediated by functional SARS-CoV-2-specific CD4+ and CD8+ memory T cells [6], leading to some concerns that these might induce HIV transcription, expression, and result in increased plasma viral load.

Studies about dynamics of pVL and HIV reservoir in individuals receiving ART following COVID-19 mRNA vaccination have been reported. Some showed increased HIV viremia, though other studies did not observe such effects [7, 8]. Some reported that COVID-19 mRNA could not induce changes in HIV reservoir size [9, 10]. For inactivated COVID-19 vaccine, which is different from mRNA vaccine, the information has been very limited.

Given these concerns, we performed a longitudinal observational study to explore whether inactivated COVID-19 vaccines could increase pVL and affect HIV reservoirs size.

## Materials and methods

### Study design

This prospective cohort study was conducted from March 2021 to February 2022. Participants were recruited from an HIV Voluntary Counseling and Testing Clinic upon obtaining written informed consent. The study design included follow-up visits at five key timepoints: before the first vaccine dose (baseline), and at 21 days after the first dose, 28 days after the second dose, six months after the second dose, and 14 days after the third dose. Venous blood samples were collected at each of these visits.

## Study population

PLWH who met the following inclusion criteria but not exclusion criteria were included in this study. Inclusion criteria: (1) No history of SARS-CoV-2 infection nor history of exposure to people diagnosed with COVID-19. (2) Willing to receive inactivated COVID-19 vaccines. Excluded criteria: (1) Participants were pregnant or lactating or had a planned pregnancy. (2) Participants with a history of allergic reaction to vaccine ingredients or additives. (3) Participants with severe or active infectious disease within last month. (4) Participants were treated with immunosuppressive drugs. (5) Participants who had been vaccinated with another vaccine within 28 days before study, or are scheduled to be vaccinated during the study period.

### Data collection and blood sample collection

Questionnaires were collected from all the enrollees on the same day they got vaccination (supplementary 1). Information including demographic characteristics, clinical history (BMI, CD4+ T cell count, duration of ART) and HIV history (HIV infection duration, baseline and nadir CD4+ T cell count) were collected. Follow-up appointments and blood sample collection were set on the same day before the first shot of vaccination (BC1), 21 days after receiving the first dose (BC2), 28 days after the second dose (BC3), six months after the second dose (BC4) and 14 days after the third shot (BC5) to collect EDTA-anticoagulated venous blood samples to test pVL, CD4+ T cell count. Moreover, venous blood samples were used to isolate peripheral blood mononuclear cells (PBMCs) by density gradient centrifugation (Lymphoprep, Stemcell, Germany). Isolated PBMCs were frozen in liquid nitrogen, according to standard procedures, until use. PBMCs were resuscitated in culture medium (RPMI 1640, 10% FCS, 2mmol/liter L-glutammine) at 37℃ without culture before nucleic acids were extracted to test total HIV-1 DNA. This study was in compliance with the Helsinki Declaration and was approved by the Human Research Ethics Committee of Beijing CDC (Ethical approval No. 2021-06).

### CD4 + T cell count and HIV viral load testing

BD FACSCanto™ and TruCount microsphere kit (BD, Milpitas, USA) was used to detect the number and proportion of CD4+ T cells from EDTA-anticoagulated venous blood. HIV Viral load in plasma was tested using COBAS Taqman HIV-1 Qualitative test kit (Roche, Mannheim, Germany). The limit of quantitation of this assay was 20 copies/ml (cp/ml).

### Total HIV-1 DNA quantitation

Total HIV-1 DNA was extracted from freshly resuscitated PBMCs without culture using Nucleic Acid Extraction Kit (SUPBIO, Guangzhou, China). The number of cells used for HIV-1 DNA detection ranged from 0.76*10^6^ to 6.8*10^6^, higher than the minimum number of cells (2*10^5^) required by the quantitative detection kit. We used SUPBIO total HIV-1 DNA Quantitative PCR Kit (SUPBIO, Guangzhou, China) quantitating total HIV-1 DNA and cell number simultaneously, following the manufacturer’s instructions. In brief, we took 200 µL of HIV DNA weak positive QC, strong positive QC, negative QC and clinical specimens respectively, and carried out the genomic DNA extraction operation. Prepared HIVDNA PCR reaction reagent at a ratio of 29.2 µL of HIV DNA PCR reaction solution and 0.8 µL of enzyme system per sample. The HIV DNA quantitative references were diluted with a 10-fold gradient of diluent and labeled as HIV DNA quantitative reference 1 to 4, respectively. 20µL of the specimen, HIV DNA quantitative reference 1~ 4, strong QC, weak QC and negative QC were added to the prepared PCR tubes, respectively. PCRs were performed under the following conditions: 5min at 37℃, 10 min at 94◦C; 5 cycles of 15s at 95◦C, 15s at 65◦C and 20s at 72◦C; 10 cycles of 15s at 95◦C, 15s at 62◦C and 20s at 72◦C; 40 cycles of 15s at 95◦C, 15s at 52◦C and 32s at 72◦C (fluorescence acquisition). In the same specimen, two standard curves were used to quantify HIV-1 DNA and nucleated cells, respectively. The result of HIV-1 DNA quantification (copies/µL) was divided by the result of nucleated cells quantification (cells/µL) and multiplied by 1 × 10^6^ to obtain the amount of HIV-1 DNA per 1 × 10^6^ nucleated cells in the specimen. The linear quantification range of the SUPBIO total HIV-1 DNA quantitative kit was 20 copies/10^6^ PBMCs to 100,000 copies/10^6^ PBMCs. Total HIV-1 DNA quantitation of all the samples collected at different time points in this study was performed on the same day using the same equipment and the same batch of reagents.

### Anti- RBD IgG antibody testing

Chemiluminescent immunoassay (CLIA) using COVID-19 virus kits (2019-nCoV IgG antibody detection kit; Bioscience Diagnostics, Tianjin, China) was carried out with plasma samples collected at five time points to detect COVID-19 IgG antibodies at different time points as described in a previous published work [1].

### Vaccines used in this study

We used two inactivated COVID-19 vaccines in this study, CoronaVac (SinoVac, Beijing, China) and Covilo (Sinopharm, Beijing, China). CoronaVac contains 600SU inactivated SARS-CoV-2 antigen from the CZ02 strain grown in Vero, aluminum hydroxide as an adjuvant, and 0.5mL per injection. Covilo was prepared using the SARS-CoV-2 19nCoV-CDC-Tan-HB02 strain grown in Vero, contains 6.5U inactivated SARS-CoV-2 antigen, aluminum hydroxide as an adjuvant, and 0.5mL per injection.

### Statistical analysis

The data were analyzed using IBM SPSS Statistics 20.0 (IBM Corp). An analysis of baseline characteristics was performed using median (interquartile range [IQR]) or frequency. Undetectable viral load was assigned a level of 10 cp/ml. Viral load with < 20 cp/ml was assigned as 15 cp/ml. Friedman test was used to compare the difference in the viral loads or total HIV-1 DNA among related samples at five time points. Pairwise comparison was performed by Friedman multiple comparison test with Bonferroni correction. Mann-Whitney U test was used to compare the difference in the total HIV-1 DNA between group of nadir CD4+ T ≤ 300 and group of nadir CD4+ T > 300. We analyzed the correlation between HIV-1 DNA and age, duration of HIV infection, and RBD-specific IgG using spearman correlation. Categorical variables were analyzed using the Chi-squared test, or Fisher’s exact test when the frequency was less than five in one or more conditions.

## Results

### Study population and recruitment

This observational study followed a convenience sample of people living with HIV (PLWH), with its design adapting to the China’s evolving COVID-19 vaccination guidelines during the COVID-19 pandemic. Initially, 80 participants on antiretroviral therapy were enrolled under the two-dose regimen for longitudinal follow-up, which involved three scheduled blood collections: baseline (BC1), three weeks post-first dose (BC2), and four weeks post-second dose (BC3). Eight participants were lost to follow-up due to travel restrictions, leaving 72 who completed this phase. In October 2021, a third vaccine dose was nationally recommended for high-risk groups. We conducted an additional follow-up in November 2021. At this time, 47 previously enrolled HIV-positive participants had completed the three-dose regimen, while 25 remained unvaccinated, forming our final study cohort. From 25 unvaccinated participants, EDTA-anticoagulated venous blood samples were collected at five timepoints (BC1-BC5) for further analysis.

### Demographic characteristics, clinical history and HIV history

A total of 25 HIV-infected individuals were enrolled in the research. An overview of the demographic characteristics, clinical history and HIV history can be found in Table 1. The median age of the subjects was 34.0 (IQR 28.5 − 40.0) years old, and most of them were males (92%, 23/25).

**Table 1:** Participant characteristics, clinical history and HIV history

Characteristic	*N* = 25
Age at enrollment in years, median (IQR)	34.0(28.5, 40.0)
Gender	
Male, n (%)	23(92.0%)
Female, n (%)	2(8%)
Low nadir CD4+ T-cell count in cells/mm3, median (IQR)	300 (198–415)
Nadir CD4+ T-cell count ≤ 300	200.0(37.5–270.0)
Nadir CD4+ T-cell count > 300	415.0(366–536.0)
Baseline CD4+ T-cell count in cells/mm3, median (IQR)	461.9 (360.1–565.7)
Duration years of HIV infection (IQR)	5.3 (3.8 − 7.3)
Years on ART, median (IQR)	5.1(3.7–6.7)
Current ART regimen	
NRTI and NNRTI	22 (88.0%)
NRTI and integrase inhibitor	2(8.0%)
Protease inhibitors and NNRTI	1 (4.0%)
Inactivated COVID-19 vaccine regimen	
Sinovac-CoronaVac	23 (92.0%)
Sinopharm-Covilo	2 (8.0%)

Among 25 HIV-infected individuals, the median duration of HIV infections and ART taking was 5.3 (IQR 3.8–7.3) years and 5.1 (IQR 3.7–6.7) years respectively. All participants had been receiving ART at enrollment. Among the 25 HIV-infected individuals, 88% (22/25) used state-funded free drugs which included two nucleotide reverse transcriptase inhibitors (NRTIs) and one non-NRTIs (NNRTIs), 4% (1/25) used protease inhibitors with two NRTIs and 8% (2/25) used integrase inhibitors with one or two types of NRTIs. 52% (13/25) of enrolled participants had a nadir CD4+ T cell counts less than or equal to 300 cells/mm^3^. Median count of the nadir CD4+ T-cell was 300 (IQR 198–415; range 8–653) cells/mm^3^.

All the participants received three doses of the inactivated SARS-CoV-2 vaccines. EDTA-anticoagulant venous blood from 25 participants were collected from HIV-infected individuals at prevaccination (Day 0), 21 days after the first dose of COVID-19 vaccine, 28 days after the second dose, 6 months after the second dose, and 14 days after the third dose, respectively. All the participants remained COVID-19 naive throughout follow-up.

At the baseline (prevaccination) visit, the most recent CD4+ T-cell count bef**ore** vaccination was 461.9 (IQR 360.1-565.7; range 143.3–782.0) cells/mm^3^. 19 (76.0%) of participants had pVL undetectable, and four (16.0%) below the lower limit of quantitation of 20 cp/ml. The highest pVL observed at baseline was 105 cp/ml.

Two inactivated COVID-19 vaccines were used in this study, CoronaVac (SinoVac) and Covilo (Sinopharm). Two (8.0%) of participants received three doses of Covilo (Sinopharm) and 23(92.0%) received three doses of CoronaVac (SinoVac).

### Dynamic changes in pVL before and after vaccination

A total of 25 HIV-infected individuals were followed up five times through the whole program. HIV pVL testing was performed at the baseline visit (prevaccination), 20 days (IQR: 19 − 21) after first vaccine dose, 27 days (IQR: 26 − 28) after the second dose, 187 days (IQR: 180 − 192) after second dose and 14 days (IQR: 13 − 15) after the third dose.

pVL < 2E + 1 was defined as 15 cp/ml. Undetectable pVL was defined as 10 cp/ml. Median value of pVL were 10 (IQR: 10-12.5), 10 (IQR: 10–10), 10 (IQR: 10–10), 10 (IQR: 10–15) and 10 (IQR:10-12.5) at each sampling time points of BC1, BC2, BC3, BC4 and BC5, respectively. Friedman test was performed to analyze the difference of pVL at various time points. No significant difference in pVL was observed among five time points (*p* = 0.105).

pVL of fifteen HIV-infected individuals (60%, 15/25) were undetectable at BC1, BC2, BC3, BC4 and BC5. Three HIV-infected persons (12%, 3/25) had pVL consistently below 20 copies at all five time points. Seven HIV-infected persons (28%, 7/25)had pVL tests of more than 20 copies on at least one of five occasions, with five (20%, 5/25) had pVL consistently below 50 and the remaining two had pVL higher than 50 at least once out of five.

Kinetics of pVL before and after each dose vaccine at five time points was shown in Table 2. At baseline, the pVL of nineteen (76.0%, 19/25) participants were undetectable, four (16.0%, 4/25) had a pVL < 20 cp/ml, and two (8.0%, 2/25) had a pVL ≥ 20 cp/ml with the highest value of 105 cp/ml. Three weeks after the first vaccine dose, 20 (80%, 20/25) participants had undetectable pVL, three (12.0%, 3/25) were < 20 cp/ml and two (8.0%, 2/25) had a pVL of ≥ 20 cp/ml. Four weeks after the second dose, 23 (92%, 23/25) had undetectable pVL and three (8.0%, 2/25) had pVL < 20 cp/ml. About six months after the second dose, 17 (68%, 17/25) were undetectable, three (12%, 3/25) had pVL < 20 cp/ml, and five (20%, 5/25) had pVL ≥ 20 cp/ml with the highest value of 130 cp/ml. Two weeks after the third dose, 19 (76%, 19/25) were undetectable, four (16%, 4/25) had pVL < 20 cp/ml, and two (8.0%, 2/25) had pVL ≥ 20 with the highest value of 134 cp/ml. No significant difference in the proportion of pVL groups was observed among time points BC1, BC2, BC3, BC4 and BC5 (*p* = 0.506), respectively.

**Table 2:** Characteristics of pVL dynamics before and after vaccination

Time points	Low nadir CD4+ T ≤ 300 *n* (%)				Low nadir CD4+ T > 300 *n* (%)		
	Undetectable	pVL < 20	pVL ≥ 20		Undetectable	pVL < 20	pVL ≥ 20
BC1	11(84.6)	2(15.4)	0(0.0)		8(66.7)	2(16.7)	2(16.7)
BC2	10(76.9)	2(15.4)	1(7.7)		10(83.3)	1(8.3)	1(8.3)
BC3	13(100.0)	0(0.0)	0(0.0)		10(83.3)	2(16.7)	0(0.0)
BC4	10(76.9)	1(7.7)	2(15.4)		7(58.3)	2(16.7)	3(25.0)
BC5	10(76.9)	3(23.1)	0(0.0)		9(75.0)	1(8.3)	2(16.7)

### Characteristics of HIV reservoir size before and after vaccination

To determine whether receiving inactivated COVID-19 vaccine could induce changes in total HIV-1 DNA copies, we quantified the number of total proviral copies per million PBMC.

**Fig. 1:**
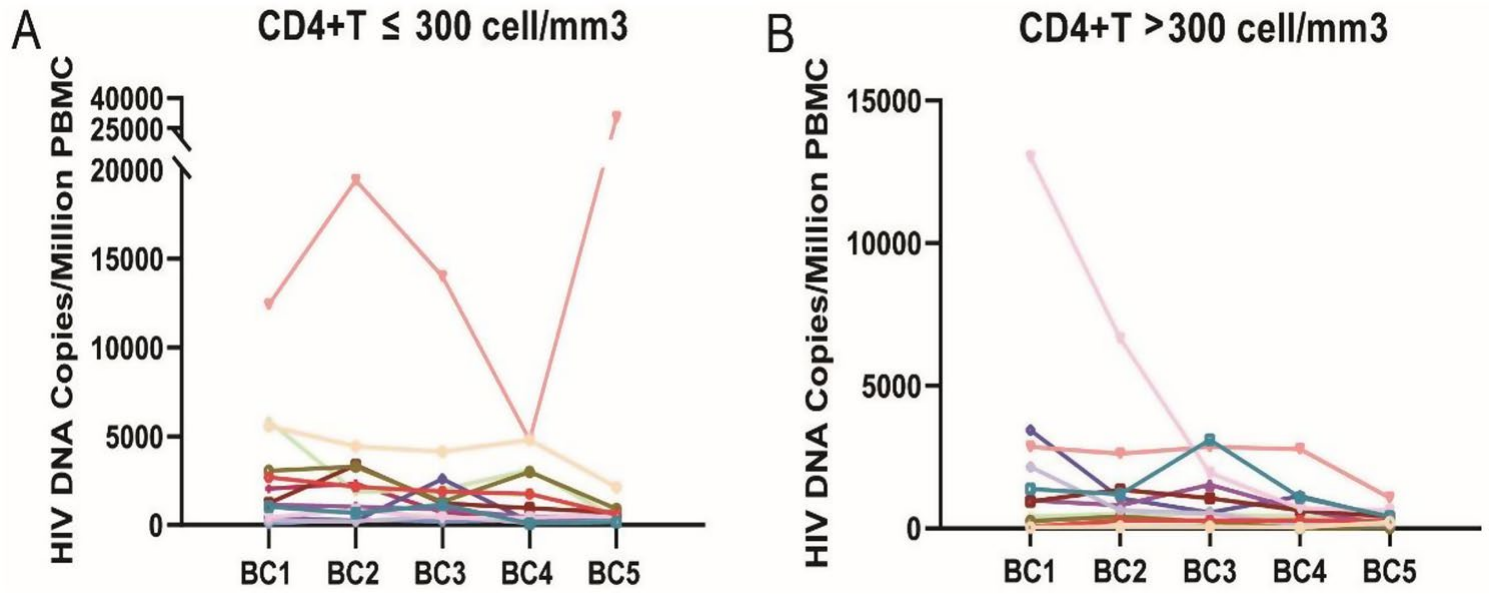
Kinetics of total HIV-1 DNA levels of each individual. A total of 25 samples were collected at each time point of BC1, BC2, BC3, BC4 and BC5. **A**: nadir CD4+ T cell ≤ 300. **B**: nadir CD4+ T cell > 300. BC1: prevaccination, BC2: three weeks after the first dose, BC3: four weeks after the second dose

A total of 25 PLWH who completed whole program were divided into two groups based on nadir CD4+ T cell (Fig. 1). Their median value of total HIV-1 DNA copies at BC1, BC2, BC3, BC4 and BC5 were listed in Table 3. Significant difference was observed in total HIV-1 DNA copies among different time points in both groups (CD4+ T cell ≤ 300 group: *p* = 0.029, CD4+ T cell > 300 group: *p* = 0.001). In the group of nadir CD4+ T cells ≤ 300, the pairwise comparison of five sets of data showed no significant difference (*P* > 0.05). In the group of nadir CD4+ T cells > 300, total HIV-1 DNA copies at BC5 was significantly lower than BC1 (*P* = 0.043) and BC3 (*P* = 0.008).

Moreover, we compared the total HIV-1 DNA between group of nadir CD4+ T ≤ 300 and group of nadir CD4+ T > 300. No significant difference was observed in total HIV-1 DNA between two groups at BC1 (*p* = 0.328), BC2 (*p* = 0.192), BC3 (*p* = 0.192) and BC4 (*p* = 0.253), respectively. While, we found total HIV-1 DNA of nadir CD4+ T > 300 was significantly lower than that in group of nadir CD4 + T ≤ 300 at BC5 (*p* = 0.019).

**Table 3:** Characteristics of total HIV-1 DNA dynamics before and after vaccination

Time points	HIV-1 total DNA copies Nadir CD4 + T ≤ 300	HIV-1 total DNA copies Nadir CD4 + T > 300	*P* ^#^
BC1	1227.2(431.6, 4307.0)	965.7(129.6, 2677.4)	0.328
BC2	1871.9(479.7, 3340.6)	717.3(307.2, 1309.9)	0.192
BC3	1241.2(606.4, 2293.8)	542.9(241.1, 1843.8)	0.192
BC4	440.9(236.4, 3041.36)	526.6(56.7, 984.9)	0.253
BC5	554.3(342.7, 819.3)	238.0(122.3, 418.2)	**0.019**
*p	**0.029**	**0.001**	

Note: * Friedman test was used to compare the difference of HIV-1 total DNA copies among BC1, BC2, BC3, BC4 and BC5. ^#^ Mann-Whitney U test was used to compare the difference in the total HIV-1 DNA between group of nadir CD4+ T ≤ 300 and group of nadir CD4+ T > 300. Bold values meant significant differences were observed between or among groups

### SARS-CoV-2-specific binding antibodies

S/CO value of IgG among different groups at different time points were showed in Table 4. On the whole, titers of RBD-specific IgG antibodies increased with each dose of vaccination. 28 days after the second dose, titers of RBD-specific IgG antibodies were enhanced to a median S/CO value of 5.832 (IQR: 1.804, 18.951) for HIV-infected with low nadir CD4+ T cell count ≤ 300 and 8.257(3.285, 12.024) for HIV-infected with low nadir CD4+ T cell count > 300, respectively. While S/CO value of each group declined at 190 days after the second dose. Fourteen days after the third vaccination, S/CO value of two groups increased considerably, with a S/CO value of 34.626 (IQR: 24.557, 70.803) in group of low nadir CD4+ T ≤ 300, and 56.004(16.784, 95195) in group of low nadir CD4+ T > 300. No significant difference was observed in S/CO value between two groups at each point.

**Table 4:** RBD-specific IgG e after each shot of COVID-19 vaccine in HIV-Infected (stratified by low nadir CD4+ T cell count) persons

Time points	S/CO value of IgG Low nadir CD4+ T ≤ 300	S/CO value of IgG Low nadir CD4+ T > 300	*p* ^#^
BC1	0.029(0.024, 0.038)	0.035(0.205, 0.0925)	0.437
BC2	0.101(0.039, 0.632)	0.548(0.165, 1.392)	0.068
BC3	5.832(1.804, 18.951)	8.257(3.285, 12.024)	1.000
BC4	1.275(0.374, 2.704)	1.155(0.800, 2.012)	0.913
BC5	34.626 (24.557, 70.803)	56.004(16.784, 95.195)	0.415

Note: ^#^ Mann-Whitney U test was used to compare the difference in the total HIV-1 DNA between group of nadir CD4+ T ≤ 300 and group of nadir CD4+ T > 300

### Association factors with copies of HIV-1 DNA before and after vaccination

Among group of nadir CD4+ T cell count ≤ 300, total HIV-1 DNA from BC1, BC2, BC3, BC4 and BC5 were closely correlated with each other. The highest correlation coefficient was observed as 0.890 between BC2 and BC5 (*P* < 0.001). The lowest was 0.566 between BC1 and BC5 (*P* = 0.044). Among group of nadir CD4+ T cell count > 300, total HIV-1 DNA from BC1, BC2, BC3, BC4 and BC5 were also closely correlated with each other. The highest correlation coefficient is 0.909 between BC3 and BC5 (*P* < 0.001). The lowest was 0.580 between BC1 and BC5 (*P* = 0.048) (supplementary table).

Among group of nadir CD4+ T cell count > 300, duration of HIV infection was positively correlated with HIV-1 DNA copies at BC4 (*R* = 0.729, *p* = 0.007) and BC5 (*R* = 0.690, *p* = 0.013). S/CO value of RBD-specific-IgG at BC3 were negatively correlated with HIV-1 total DNA copies at time points of BC2 (*R*=-0.713, *p* = 0.009) and BC3 (*R*=-0.587, *p* = 0.045). While among group of nadir CD4+ T cell count < 300, HIV-1 total DNA copies at each time point was not correlated with infection duration and S/CO of RBD-specific-IgG at each time point (supplementary table).

## Discussion

Since PLWH tend to have more serious symptoms or even death after infecting COVID-19, they were prioritized for COVID-19 vaccination in many countries [11, 12]. In this study, a longitudinal cohort study was performed on HIV-infected participants with no history of COVID-19 infection and COVID-19 vaccine inoculation. We found that receiving inactivated COVID-19 vaccine resulted in no statistically significant differences in pVL. Although there was a gradual increase in the proportion of lower pVL group after each vaccination. Significant differences were observed in total HIV-1 DNA copies among five time points in both CD4+ T cell groups, in which there was an downward trend in HIV-1 total DNA copies.

Vaccination causes a series of changes in the body, inducing neutralizing antibody responses, eliciting immune responses mediated by functional SARS-CoV-2-specific CD4 + and CD8 + memory T cells. Considering the particularities of HIV-infected individuals, whether receiving inactivated vaccine leads to pVL rebound and affects HIV reservoirs size has been a major concern for PLWH. Some studies on this topic have been performed.

For PLWH inoculated with inactivated COVID-19 vaccine, two studies reported there was no statistical changes in HIV plasma viremia after vaccination [8, 13]. , which was consistent with our result. In our study, we found an upward trend in the proportion of HIV pVL undetectable group after vaccination, although no significant difference was observed. We speculated that the killing or suppression of cells transcribing HIV by activated HIV-specific CD8+ T cells might contribute to this result [9]. Moreover, we found there was a downward trend in HIV-1 total DNA after vaccination, and HIV-1 total DNA at BC5 was significantly lower than that at BC1 and BC3 in the group of CD4+ T cells >300. These results were similar with previous studies, in which a reduction of total HIV-1 DNA in peripheral mononuclear cells was observed after vaccination [14, 15]. We speculated that vaccination might have strongly activated immune system, especially in group of PLWH with CD4+ T cell >300 and infected cells might have been eliminated by viral cytopathic effects. This phenomenon was similar with the theory of ‘shock and kill’, which involves the use of drugs that reverse latency and increase viral gene expression (shock), rendering the viral-reservoir cells vulnerable to elimination (kill) by other cells of the immune system [16]. For SARS CoV-2 mRNA vaccine, Duncan, Maggie C et al. [17] found that two doses of COVID-19 mRNA vaccines didn’t induce changes in intact proviral DNA assay. Similarly, though Stevenson et al. [13] also did not observe significant depletions of intact proviruses after two doses of mRNA vaccination, they did find significant decreases in cell-associated HIV mRNA after vaccination, suggesting killing or suppression of cells transcribing HIV. We speculated that different vaccines, different doses of vaccination program, different test target and method might have contributed to different results.

In our study, all the participants were having ART. However, when a PLWH without treatment was inoculated with inactivated COVID-19 vaccine, things have been totally different. Guang CL et al. [18] reported a case of viral activation and CD4+ T cell loss after receiving inactivated COVID-19 vaccines in a treatment-naïve HIV-positive patient. Considering that effective ART was important to control viral proliferation, we suggest vaccination should be given only to ART-suppressed individuals.

The number of CD4+ T-cell count is regarded as an indicator of severity of HIV infection. A previous study implied that PLWH with higher CD4+ T cell count produced higher titer of antibody [19]. And CD4+ T cell count in turn was negatively correlated with HIV-1 total DNA copies [20]. The above results were accordant with our study, in which IgG titer after vaccination was negatively correlated with total HIV-1 DNA copies. Moreover, we found duration of HIV infection might be positively correlated with HIV-1DNA. This was accordant with a previous article, in which after long-term of ART, the HIV-1 DNA from HIV infected individuals did not decrease but increase [21].

There were some limitations in this study. First, we did not recruit unvaccinated HIV-infected people as controls, as a result, we had no way of knowing how much HIV-1 DNA is affected by vaccination beyond the effect of the antiviral therapy regime. Second, we didn’t specifically test the level of intact proviral DNA which was associated with rapid viral rebound. Third, the sample size in this study was limited. When we do stratified analysis, the results might not be robust due to the limited sample size. And sometimes significant differences can’t be observed due to the small sample size.

## Conclusions

Although receiving inactivated COVID-19 vaccine didn’t significantly affect pVL, we observed a downward trend in HIV-1 total DNA copies after vaccination. Duration of HIV infection and IgG titer might be correlated with HIV-1 total DNA copies after vaccination.

## Supplementary Information

Below is the link to the electronic supplementary material.

Supplementary Material 1

Supplementary Material 2

## Data Availability

The datasets analyzed during the current study are not publicly available as they were collected from HIV-infected patients and are considered sensitive. Access to anonymized data will be considered on reasonable request and following approval by Beijing Center for Disease Prevention and Control.

## Abbreviations

COVID-19: Coronavirus disease 2019
HIV: Human immunodeficiency virus
PLWH: People living with HIV
IQR: Interquartile range
pVL: Plasma viral load
PBMCs: Peripheral blood mononuclear cells
ART: Antiretroviral therapy
CLIA: Chemiluminescent immunoassay
NRTIs: Nucleotide reverse transcriptase inhibitors
Non-NRTIs: Non-Nucleotide reverse transcriptase inhibitors
