# Supplementary material for: Effects of inactivated COVID-19 vaccination on HIV viremia and reservoir size: a longitudinal cohort study
Source: BMC Infect Dis. 2025 Dec 13;26:91. doi: 10.1186/s12879-025-12184-8 (PMC12821177; doi:10.1186/s12879-025-12184-8)
Supplement: Supplementary file 1 — Supplementary Material 1 [file 12879_2025_12184_MOESM1_ESM.docx]

**Questionnaire**

1. **Basic Information**
2. Name: _ _ _ _ _ _ _ _ _ _ _
3. Sex: □ Male □ Female
4. Ethnic group: _ _ _ _
5. Date of birth: MM/DD/YYYY
6. Height: _ cm; Weight: _ kg;
7. Marital status:

□ Unmarried □ Married with spouse □ Divorced or widowed

□ Cohabitation □ Unknown

1. Educational level:

□ Illiterate □ Primary school □ Junior high school

□ Senior high school or technical secondary school □ Junior college

□ Bachelor's degree □ Master's degree □ Doctor's degree

1. Household registration:

□ in this city □ in other provinces Residence: _ District, phone number: _

9. Current occupation

□ Students □Teachers □ Nurses/Nannies □ Catering/Food Industry

□ Commercial Services □ Medical Staff □ Workers □ Migrant workers

□ Farmers □ Herdsmen □ Fishermen □ Civil servants □ Company staff

□ Retired personnel □ Housework and unemployed

□ Technical personnel of enterprises and institutions □ Other _ _ _ _ _ _.

10.Current monthly income:

□ Less than 3,000 RMB □ Less than 3,000-5,000 RMB □ 5,000-10,000 RMB

□ 10,000-20,000 RMB □ 20,000-40,000 RMB □ More than 40,000 RMB

1. **Physical Health and Vaccination**
2. Whether suffering from underlying disease (such as diabetes, endocrine diseases, liver and kidney diseases, tumors, etc.):

□ No □ Yes (please specify the _ _ _ _ _ _ _ _ _ _) □ Don't know;

1. Whether infected with hepatitis B virus:

□ No □ Yes □ Don't know;

1. Whether infected with hepatitis C virus:

□ No □ Yes □ Don't know;

1. Have you ever suffered from venereal disease:

□ No □ Yes □ Don't know;

1. If you have ever suffered from venereal disease, please select below:

□ Syphilis □ Gonorrhea □ Condyloma acuminatum □ Genital herpes

□ Urethral Chlamydia trachomatis infection □ Other ___

1. Have you used glucocorticoids in the last month:

□ No □ Yes □ Don't know;

1. Did you use immunomodulator in the last month:

□ No □ Yes □ Don't know;

1. Whether contacted with COVID-19 infected persons:

□ No □ Yes (please specify the time _ _ _ _ _ _ _);

1. whether infected with COVID-19:

□ No □ Yes (please specify the time _ _ _ _ _ _ _ _).

1. Are you currently sick:

□ No □ Yes (please specify _).

**III. Information on HIV testing and diagnosis**

**(HIV-infected persons need to fill in the following form)**

1. Confirmed HIV test result:

□ Positive □ Negative □ Uncertain test time: MM/DD/YY;

1. The CD4+T cell count of the first test after HIV infection before treatment was __ , and the test date was __.
2. The lowest CD4+T cell count after HIV infection was __, and the date of testing was __.
3. The result of the last CD4+T cell count was __, and the date of the test was __.
4. The result of the first viral load test after HIV infection was __, and the date of the test was__.
5. If the previous/current HIV antibody test result is positive, the possible date of infection: .
6. The cause of HIV infection (single choice):

□ History of injection drug use

□ Spouse/fixed sex with positive history

□ History of commercial heterosexual sex

□ History of non-commercial non-regular heterosexual sex

□ History of MSM

□ Plasma donation history

□ History of blood transfusion/blood products

□ Positive maternal history

□ Occupational exposure history

□ Surgical history

□ No history of high-risk behavior

□ Others (Please specify:)

1. If you have visited the doctor, you were diagnosed as:

□ HIV infection acute stage □ asymptomatic stage

□ AIDS stage □ don't know

1. Are you receiving HARRT currently?

□ No □ Yes (please specify the drug: _)

The questionnaire is over. Thank you for your participation and support!

Investigator (Signature): _ _ _

Quality Controller (Signature): _ _ _

Investigation Date: _ _ _
