# Supplementary material for: Effects of inactivated COVID-19 vaccination on HIV viremia and reservoir size: a longitudinal cohort study
Source: BMC Infect Dis. 2025 Dec 13;26:91. doi: 10.1186/s12879-025-12184-8 (PMC12821177; doi:10.1186/s12879-025-12184-8)
Supplement: Supplementary file 2 — Supplementary Material 2 [file 12879_2025_12184_MOESM2_ESM.docx]

Supplementary table: Correlation analysis between age, duration of HIV infection, IgG and HIV-1 DNA copies

| Groups | Factors |  | HIV-1 DNA | | | | |  |
| --- | --- | --- | --- | --- | --- | --- | --- | --- |
|  |  |  | BC1 | BC2 | BC3 | BC4 | BC5 | |
| CD4+T≤300 | Age | r | 0.124 | -0.201 | 0.292 | 0.052 | -0.127 | |
|  |  | p | 0.687 | 0.511 | 0.334 | 0.865 | 0.680 | |
|  | Duration of HIV infection | r | -0.143 | 0.063 | 0.019 | -0.006 | 0.085 | |
|  |  | p | 0.641 | 0.837 | 0.950 | 0.986 | 0.782 | |
|  | IgG-BC2 | r | 0.088 | -0.049 | 0.418 | 0.055 | 0.093 | |
|  |  | p | 0.775 | 0.873 | 0.156 | 0.859 | 0.762 | |
|  | IgG-BC3 | r | 0.203 | -0.066 | 0.538 | 0.121 | 0.027 | |
|  |  | p | 0.505 | 0.831 | 0.058 | 0.694 | 0.929 | |
|  | IgG-BC4 | r | -0.055 | -0.330 | 0.192 | -0.170 | -0.352 | |
|  |  | p | 0.859 | 0.271 | 0.529 | 0.578 | 0.239 | |
|  | IgG-BC5 | r | -0.176 | -0.401 | -0.055 | -0.104 | -0.280 | |
|  |  | p | 0.566 | 0.174 | 0.859 | 0.734 | 0.354 | |
|  | BC1 | r | 1.000 | 0.841 | 0.786 | 0.868 | 0.714 | |
|  |  | p | - | **<0.001** | **0.001** | **<0.001** | **0.006** | |
|  | BC2 | r | 0.841 | 1.000 | 0.566 | 0.846 | 0.890 | |
|  |  | p | **<0.001** | - | **0.044** | **<0.001** | **<0.001** | |
|  | BC3 | r | 0.786 | 0.566 | 1.000 | 0.654 | 0.621 | |
|  |  | p | **0.001** | **0.044** | - | **0.015** | **0.024** | |
|  | BC4 | r | 0.868 | 0.846 | 0.654 | 1.000 | 0.797 | |
|  |  | p | **<0.001** | **<0.001** | **0.015** | - | **0.001** | |
|  | BC5 | r | 0.714 | 0.890 | 0.621 | 0.797 | 1.000 | |
|  |  | p | **0.006** | **<0.001** | **0.024** | **0.001** | - | |
| CD4+T >300 | Age | r | 0.277 | 0.158 | 0.378 | 0.637 | 0.361 | |
|  |  | p | 0.384 | 0.625 | 0.225 | **0.026** | 0.249 | |
|  | Duration of HIV infection | r | 0.375 | 0.532 | 0.557 | 0.729 | 0.690 | |
|  |  | p | 0.230 | 0.075 | 0.060 | **0.007** | **0.013** | |
|  | IgG-BC2 | r | 0.490 | 0.559 | -0.713 | 0.462 | 0.392 | |
|  |  | p | 0.106 | 0.059 | **0.009** | 0.131 | 0.208 | |
|  | IgG-BC3 | r | 0.462 | 0.413 | -0.587 | 0.524 | 0.399 | |
|  |  | p | 0.131 | 0.183 | **0.045** | 0.080 | 0.199 | |
|  | IgG-BC4 | r | 0.189 | 0.364 | 0.392 | 0.343 | 0.441 | |
|  |  | p | 0.557 | 0.245 | 0.208 | 0.276 | 0.152 | |
|  | IgG-BC5 | r | 0.126 | 0.245 | 0.434 | 0.308 | 0.420 | |
|  |  | p | 0.697 | 0.443 | 0.159 | 0.331 | 0.175 | |
|  | BC1 | r | 1.000 | 0.853 | 0.790 | 0.776 | 0.580 | |
|  |  | p | - | **<0.001** | **0.002** | **0.003** | **0.048** | |
|  | BC2 | r | 0.853 | 1.000 | 0.909 | 0.818 | 0.776 | |
|  |  | p | **<0.001** | **-** | **<0.001** | **0.001** | **0.003** | |
|  | BC3 | r | 0.790 | 0.909 | 1.000 | 0.874 | 0.706 | |
|  |  | p | **0.002** | **<0.001** | **-** | **<0.001** | **0.010** | |
|  | BC4 | r | 0.776 | 0.818 | 0.874 | 1.000 | 0.762 | |
|  |  | p | **0.003** | **0.001** | **<0.001** | **-** | **0.004** | |
|  | BC5 | r | 0.580 | 0.776 | 0.706 | 0.762 | 1.000 | |
|  |  | p | **0.048** | **0.003** | **0.010** | **0.004** | - | |

Note：Spearman correlation was used to analyze the correlation between factors and HIV-1 DNA. Bold Bold values meant significant differences were found between groups.
